# Exosomal Expression of CXCR4 Targets Cardioprotective Vesicles to Myocardial Infarction and Improves Outcome after Systemic Administration

**DOI:** 10.3390/ijms20030468

**Published:** 2019-01-22

**Authors:** Alessandra Ciullo, Vanessa Biemmi, Giuseppina Milano, Sara Bolis, Elisabetta Cervio, Emanuel Tudor Fertig, Mihaela Gherghiceanu, Tiziano Moccetti, Giovanni G. Camici, Giuseppe Vassalli, Lucio Barile

**Affiliations:** 1 Cellular and Molecular Cardiology Laboratory, Cardiocentro Ticino Foundation, 6900 Lugano, Switzerland; alessandra.ciullo@cardiocentro.org (A.C.); vanessa.biemmi@cardiocentro.org (V.B.); giuseppina.milano@cardiocentro.org (G.M.); sara.bolis@cardiocentro.org (S.B.); betta.cervio@gmail.com (E.C.); tiziano.moccetti@cardiocentro.org (T.M.); giuseppe.vassalli@cardiocentro.org (G.V.); 2 Molecular Cardiology Institute, Department of Cardiology, University of Zürich, 8952 Schlieren, Switzerland; giovanni.camici@uzh.ch; 3 Laboratory of Cardiovascular Research, Lausanne University Hospital, 1011 Lausanne, Switzerland; 4 “Victor Babes” National Institute of Pathology, 050096 Bucharest, Romania; fertig.e@gmail.com (E.T.F.).; mgherghiceanu@yahoo.com (M.G.); 5 Faculty of Biomedical Science, University of Italian Switzerland, 6900 Lugano, Switzerland

**Keywords:** exosomes, CXCR4, cardiac progenitor cells, intravenous injection

## Abstract

Cell therapy has been evaluated to enhance heart function after injury. Delivered cells mostly act via paracrine mechanisms, including secreted growth factors, cytokines, and vesicles, such as exosomes (Exo). Intramyocardial injection of cardiac-resident progenitor cells (CPC)-derived Exo reduced scarring and improved cardiac function after myocardial infarction in rats. Here, we explore a clinically relevant approach to enhance the homing process to cardiomyocytes (CM), which is crucial for therapeutic efficacy upon systemic delivery of Exo. By overexpressing exosomal CXCR4, we increased the efficacy of plasmatic injection of cardioprotective Exo-CPC by increasing their bioavailability to ischemic hearts. Intravenous injection of Exo^CXCR4^ significantly reduced infarct size and improved left ventricle ejection fraction at 4 weeks compared to Exo^CTRL^ (*p* < 0.01). Hemodynamic measurements showed that Exo^CXCR4^ improved dp/dt min, as compared to Exo^CTRL^ and PBS group. In vitro, Exo^CXCR4^ was more bioactive than Exo^CTRL^ in preventing CM death. This in vitro effect was independent from SDF-1α, as shown by using AMD3100 as specific CXCR4 antagonist. We showed, for the first time, that systemic administration of Exo derived from CXCR4-overexpressing CPC improves heart function in a rat model of ischemia reperfusion injury These data represent a substantial step toward clinical application of Exo-based therapeutics in cardiovascular disease.

## 1. Introduction

Over the past 20 years, cell-therapy approaches have been investigated as an exciting strategy to repair heart tissue after myocardial infarction (MI) [1,2,3]. Today the prevailing view is that stem and progenitor cells elicit their therapeutic effects via indirect paracrine mechanisms that result in the salvage of myocardium at risk, rather than cardiac regeneration by trans-differentiating into cardiomyocytes (CM) [1,4]. This notion implies that therapeutic agents, including cells and cell-derivatives, such as extracellular vesicles (EV), act within the context of cardioprotection, and it would ideally be delivered as early as possible after acute MI [4]. In patients after acute MI, intracoronary (IC) or systemic delivery of bio-therapeutics at very early time points, soon after the percutaneous coronary angioplasty (for instance, as it has been implemented with systemic thrombolysis) [5], would be desirable to achieve cardioprotection, while also minimizing the risk of intramyocardial (IM) injections using a percutaneous catheter. 

Recently data pointed to exosomes (Exo), nanosized EV formed into cytosolic sub-compartments, as the main indirect mechanism of benefit of cardiac progenitor cells (CPCs) and other cell types, such as bone marrow-derived mesenchymal stem cells (MSCs), into infarcted hearts [6,7,8,9,10]. Exo are composed of a phospholipidic bilayer that encloses proteins, nucleic acids (mRNA, DNA, microRNA), and lipids [11,12], which mediate a variety of intercellular signals [13,14]. In a porcine model of chronic MI, Exo-CPC were shown to promote neovascularization and to decrease infarct size following IM, but not IC administration [15]. It was hypothesized that these differences between the two routes of administration might be due to limited Exo retention in the heart after IC delivery [15]. This raises the question on how to enhance Exo homing to injured myocardium.

CXCL12 (also known as stromal cell-derived factor 1; SDF-1α) is a member of the CXC chemokine family, which is overexpressed in ischemic tissues [16,17], including infarcted myocardium. The expression of this chemokine in infarcted myocardium is upregulated because the activation of hypoxia-induced factor-1(HIF-1) [18]. It has been shown that SDF-1α plasma levels are increased in patients post-MI [17]. SDF-1α binds the CXCR4 receptor, acting as a potent chemoattractant for CXCR4 expressing cells, including circulating progenitor cells [19]. This effect by SDF-1α play a role in cardiac repair through the recruitment of circulating progenitor cells to the infarct zone [20,21]. To maximize the effect of SDF-1α for tissue regeneration, several groups have genetically engineered MSCs to overexpress CXCR4, which resulted in enhanced MSC mobilization, engraftment, and cardiac repair in vivo [22,23,24]. Kang K et al. showed that using an in vivo rat MI model, a cell sheet of MSCs primed with Exo overexpressing CXCR4 (Exo^CXCR4^) was able to preserve heart function when applied soon after coronary artery ligation, whereas the MSC sheet exposed to control Exo was ineffective. These results indicated that MSC-derived Exo^CXCR4^ enhance MSC-mediated cardioprotection [25]. The in vivo direct effect of Exo^CXCR4^ by intravenous injection has not yet been tested. In our design, we propose a systemic delivery of Exo rather than Exo-mediated priming of injectable cells. To address this question, we engineered CPCs to overexpress CXCR4 (CPC^CXCR4^). Although CPC share with MSC the expression of well-known surface markers, such as CD105, CD90, CD73, Exo secreted by CPC resulted deeply different in terms of protein content and functional properties compared to Exo-MSC [6]. Having recently described the cardioprotective activities of IM injected Exo-CPC in a rat MI model [6], here we explored whether CPC^CXCR4^ –derived Exo exhibit superior homing and cardioprotective effects, as compared to naturally occurring Exo injected via tail-vein. We hypothesized that the formation of an SDF-1α gradient towards the heart following MI might increase the bioavailability and the retention of Exo^CXCR4^ within the tissue. Particularly, we evaluated the therapeutic efficacy of systemic Exo^CXCR4^ administration in an acute model of ischemia/reperfusion (I/R). I/R model has recently been indicated as the most appropriate preclinical model for studying cardioprotective strategies [26]. Additionally Exo homing to, and uptake by, CM was studied ex vivo in isolated-perfused hearts. Finally, we also sought to confirm their cardioprotective properties in human iPS-derived CM.

## 2. Results

### 2.1. CPC^CXCR4^ Load Exogenous Receptor into Exo at Protein and mRNA Levels

**Figure 1:**
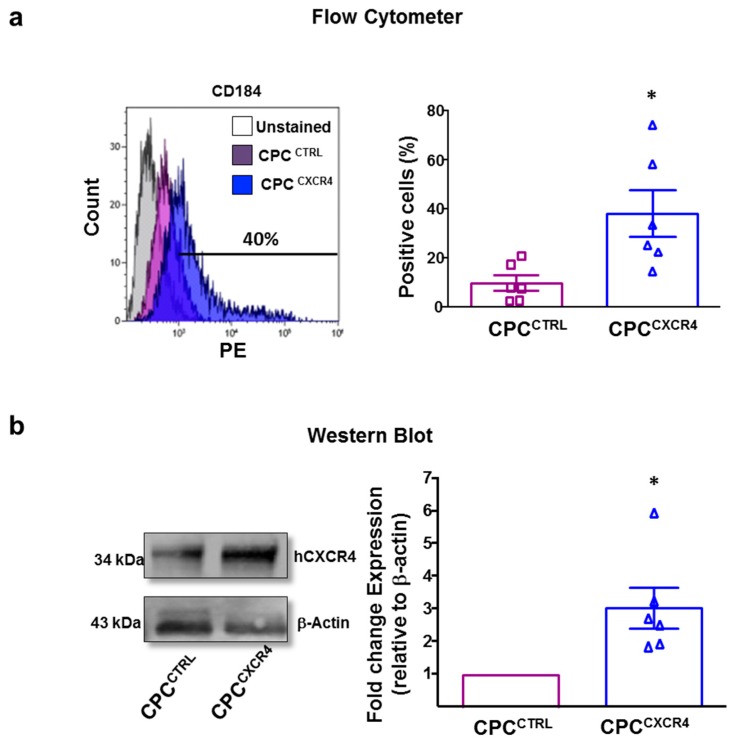
The hCXCR4 expression. (**a**) Flow cytometer analyses for relative expression of hCXCR4 (CD184) in control CPC (CPC^CTRL^, purple histogram), and CXCR4 overexpressing CPC (CPC^CXCR4^, blue histogram). Quantitative data are percentage of positive cells (*n* = 6 patients/group) * *p* < 0.05 (two-tailed, paired Student *t*-test). (**b**) Western blot showing expression of CXCR4 in CPC^CTRL^ vs. CPC^CXCR4^. Quantitative data are relative expression levels (densitometry) of CXCR4 normalized for β-actin and showed as fold-changes to CPC^CTRL^ (*n* = 6 patients/group) * *p* < 0.05 (two-tailed, paired Student *t*-test).

**Figure 2:**
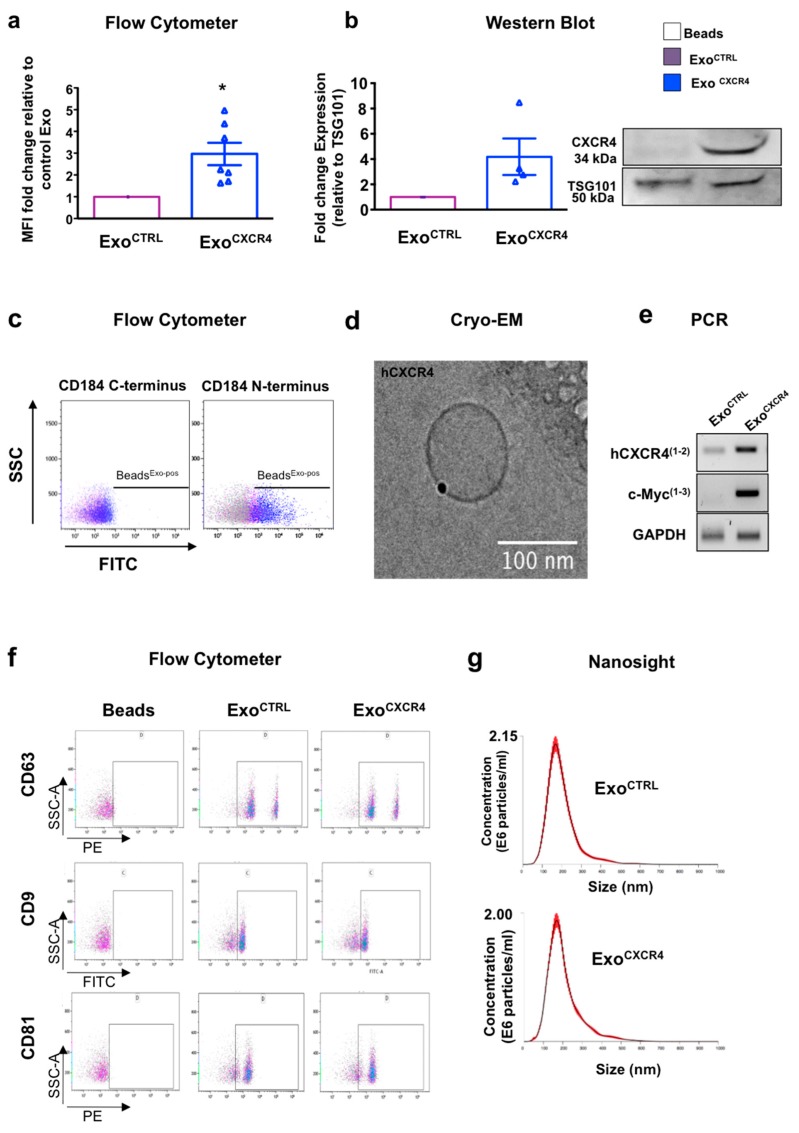
CXCR4 expression. (**a**) Quantitative data flow cytometer analyses for relative expression of CXCR4 in Exo^CTRL^ (purple bar), and CXCR4 overexpressing Exo (Exo^CXCR4^, blue bar). Exo were bound to beads before analyses as described in the method section. Data are fold-changes in mean fluorescence intensity (MFI) versus controls (*n* = 7 patients/group) * *p* < 0.05 (two-tailed, paired Student *t*-test). (**b**) Western blot showing expression of CXCR4 in Exo^CXCR4^ vs. Exo^CTRL^. Quantitative data are relative expression levels of CXCR4 normalized for TSG101 and showed as fold-changes to Exo^CTRL^ (*n* = 4 patients/group). (**c**) Bead-based flow cytometric analysis for Exo. Two different antibodies directed against the N-terminus (extraluminal-domain) and the C-terminus (intraluminal-domain) region of hCXCR4 (CD184) were used in the absence of permeabilization. Only the anti-N-terminus CXCR4 antibody resulted in a positive staining, as shown by the right-shift of bead-Exo complex. (**d**) Cryo-EM image of Exo^CXCR4^ immunolabeled with rabbit CXCR4 as a primary Ab, and goat-anti-Rabbit IgG 25 nm gold conjugate as a secondary Ab, showing CXCR4 on the surface of a particle. (**e**) The hCXCR4-mRNA expression. Conventional RT-PCR showing the presence of hCXCR4 mRNA in Exo. The exogenous sequence (c-Myc) is only present into Exo^CXCR4^. Primers used for individual PCR assays, are indicated by numbers in brackets in the Appendix Figure A1. (**f**) Expression of the exosome markers, CD9, CD63, and CD81, as assessed by flow cytometry in Exo^CTRL^ purple dots), and Exo^CXCR4^ (blue dots). (**g**) Size distribution of Exo. Averaged Size/Concentration of Exo^CTRL^ (*n* = 10 repeated measurements of 3 different patients) and Exo^CXCR4^ (*n* = 10 repeated measurements of 3 different patients). Red error bars indicate +/− SE of the mean.

**Figure A1:**
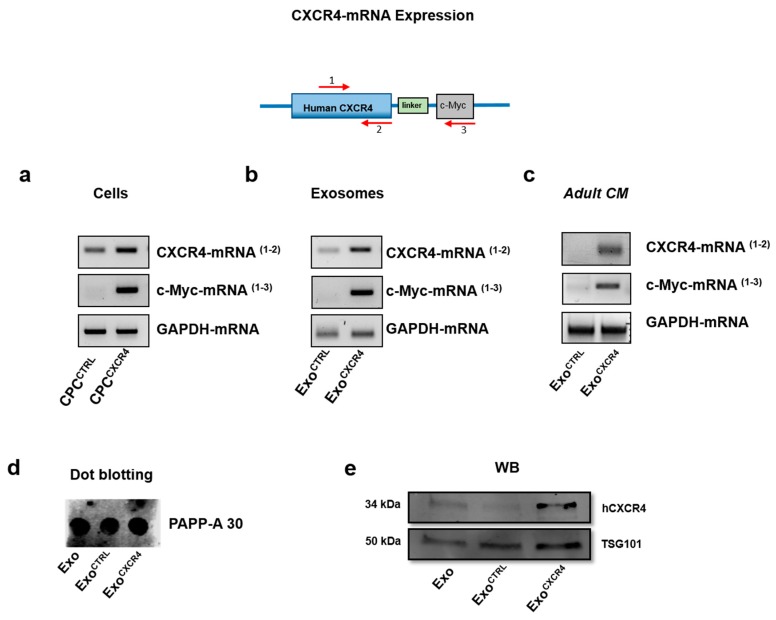
CXCR4-mRNA expression. (**a**) Conventional RT-PCR showing the presence of CXCR4 mRNA in cells before and after transfection (CPC^CTRL^ vs. CPC^CXCR4^). The exogenous sequence (c-Myc) is only present into transfected cells (CPC^CXCR4^). (**b**)The hCXCR4-mRNA is slightly present in Exo^CTRL^ and its expression increases in Exo produced by transfected cells (Exo^CXCR4^). The sequence of c-Myc tag is present in Exo^CXCR4^. (**c**) Representative PCR analyses on isolated adult CM from ex vivo experiments; the sequence of c-Myc tag is present in Exo^CXCR4^. Primers used for individual PCR assays are indicated by numbers in brackets, as for the top drawing. (**d**) Representative dot immunoblotting using 4PD4-PAPP30 mAb that selectively detects dimeric (active) PAPP-A, which was unchanged in naïve Exo, Exo derived from cell transfected with backbone plasmid (Exo^CTRL^) and Exo derived from cells overexpressing hCXCR4 (Exo^CXCR4^ ). (**e**) Representative western blotting analysis showing level of expression in Exo, Exo^CTRL^ and Exo^CXCR4^.

Although primary naïve CPC express the transmembrane receptor CXCR4 (CD184), this is poorly expressed on the surface of Exo secreted by these cells. Therefore, we used PCMV3-Cx-Myc plasmid in an attempt at enriching cells and Exo for hCXCR4 receptor. Transfection significantly increased expression of CXCR4 in cells (CPC^CXCR4^) in comparison to CPC transfected with empty backbone (CPC^CTRL^), as verified by flow-cytometer (FC) and western blot (WB) analyses (Figure 1a,b). CXCR4 could be detected on Exo secreted by CPC^CXCR4^ (Exo^CXCR4^) and slightly onto surface of Exo derived from CPC^CTRL^ (Exo^CTRL^) (Figure 2a,b). The level of expression of CXCR4 in Exo^CTRL^ was comparable to naïve expression of this receptor with no increment due to the transfection of backbone plasmid (Figure A1e).

To verify the correct transmembrane orientation of the receptor in Exo^CXCR4^, FC analysis was performed using two different antibodies directed against both the N-terminus (extraluminal-domain) and the C-terminus (intraluminal-domain) region in the absence of permeabilization. Only the anti-N-terminus CXCR4 antibody resulted in a positive staining (Figure 2c). Data was confirmed by Cryo Electron Microscopy (Cryo-EM) analysis and immunogold staining experiment, using anti-CXCR4 (N-terminus) as a primary antibody (Millipore) (Figure 2d).

In order to determine the presence of endogenous versus exogenous CXCR4 mRNA in Exo, we constructed 2 primer pairs that specifically amplified the hCXCR4 sequence only (pairs 1,2, Figure A1) and the hCXCR4 sequence with the fused c-Myc tag (pairs 1–3, Figure 2e). The hCXCR4 mRNA was slightly detectable in Exo^CTRL^ and dramatically increased in Exo^CXCR4^. Only Exo^CXCR4^ contained the exogenous sequence as evidenced by the presence of the c-Myc tag amplicon (Figure 2e). By WB analysis, Exo^CTRL^ and Exo^CXCR4^ expressed similar levels of the Exo specific marker TSG-101 (Figure 2b), and tetraspanins (CD63, CD9, CD81) by FC analysis (Figure 2f). The expression of functional pregnancy-associated plasma protein-A (PAPP-A), previously showed to be functional into Exo-CPC [6], was similar in Exo^CTRL^ and Exo^CXCR4^ (Figure A1d). Moreover, the overexpression of CXCR4 does not impair size/concentration distributions of Exo, as assessed by Nanosight analysis (Figure 2g).

### 2.2. Intravenous Injection of Exo^CXCR4^, but not Exo^CTRL^, Is Protective after In Vivo I/R Injury

**Figure 3:**
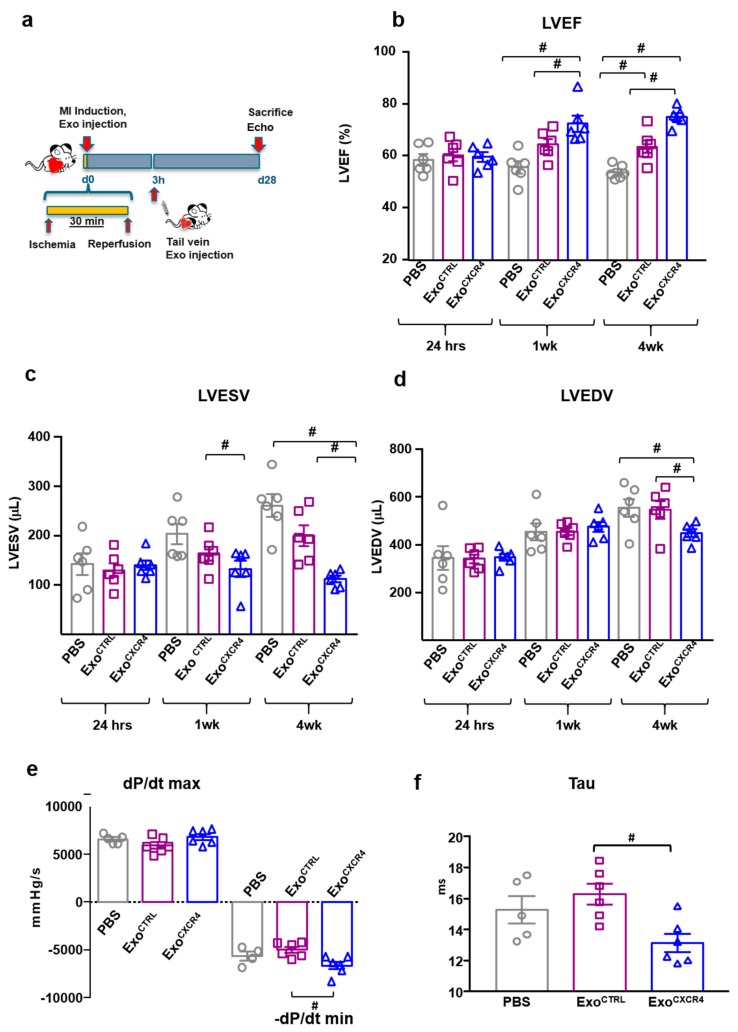
In vivo cardio-protection after I/R. (**a**) Scheme depicting the study protocol. I/R model was induced by ligating the left descending anterior coronary that was then released after 30 min. Exo (2 × 10^11^ total particles from a pool of *n* = 6 patients, in 300 μL PBS) were injected tail vein 3 h after reperfusion. (**b**) Exo^CXCR4^ but not Exo^CTRL^ significantly improved LVEF at 7days after I/R, compared to PBS control group. At 28 days after I/R, Exo^CXCR4^, and to a lesser extent Exo^CTRL^, significantly improved LVEF compared to control group (*n* = 6 rats/group), # *p* < 0.05 (one-way analyses of variance-ANOVA with post-hoc multiple comparisons using the Tukey-Kramer multiple comparisons test for paired parametric comparisons; test was performed at each time-point). (**c**,**d**) Exo^CTRL^ reduced both left ventricle (LV) end-systolic volume (LVESV) and left ventricle end-diastolic volume (LVEDV) was significantly less than modified Exo ^CXCR4^ (*n* = 6 rats/group), # *p* < 0.05 (one-way analyses of variance-ANOVA with post-hoc multiple comparisons using the Tukey-Kramer multiple comparisons test for paired parametric comparisons; test was performed at each time-point). (**e**) Hemodynamic measurements showing a decreased ventricular compliance in the Exo^CXCR4^ by quantitative analysis of –dp/dt min and dp/dt max (*n* = 6 rats/group for Exo^CTRL^ and Exo^CXCR4^; *n* = 5 rats/group PBS) # *p* < 0.05 (one-way analyses of variance-ANOVA with post-hoc multiple comparisons using the Tukey-Kramer multiple comparisons test for paired parametric comparisons; test was performed at each time-point). (**f**) From LV pressure/volume loops, the time constant of isovolumic LV pressure fall (Tau) value, which reflects diastolic relaxation properties, was calculated. LV relaxation was improved and time constant reduced in the Exo^CXCR4^ (*n* = 6 rats/group) group compared with the Exo^CTRL^ (*n* = 6 rats /group) and PBS (*n* = 5 rats/group) # *p* < 0.05 (one-way analyses of variance-ANOVA with post-hoc multiple comparisons using the Tuke–Kramer multiple comparisons test for paired parametric comparisons).

**Figure 4:**
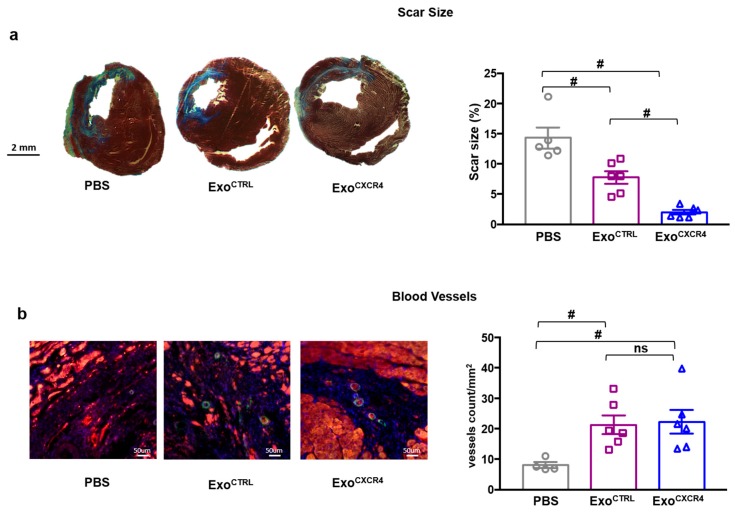
(**a**) Masson’s Trichrome staining for the quantification of infarct size. Exo reduce scar size at 28 days after I/R. The reduction in scar size is more pronounced in Exo^CXCR4^ compared to Exo^CTRL^ treated group (*n* = 5 rats/group PBS, *n* = 6 rats/group Exo^CTRL^ and Exo^CXCR4^). (**b**) Exo^CTRL^ and Exo ^CXCR4^. increased blood vessel density. SMA, smooth muscle actin (green); TnI, Troponin I (red); nuclear counterstaining (blue); *n* = 4 rats/group PBS, *n* = 6 rats/group Exo^CTRL^ and Exo^CXCR4^). # *p* < 0.05 (one-way analyses of variance-ANOVA with post-hoc multiple comparisons using the Tukey–Kramer multiple comparisons test for paired parametric comparisons).

**Figure A2:**
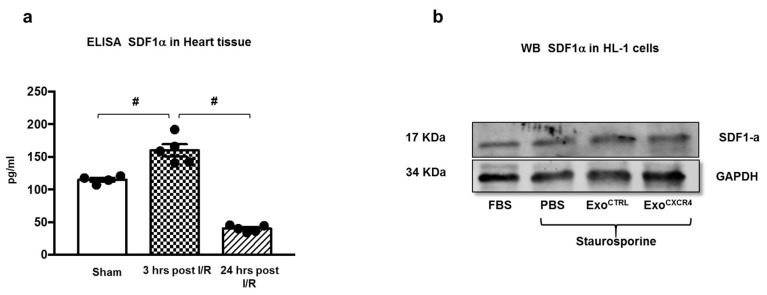
(**a**) Levels of SDF-1α detected by ELISA in rat heart tissue at 3 and 24 h post MI (*n* = 4 rats/group for Sham; *n* = 5 rats/group for 3h and 24h) # *p* < 0.05 (one-way analyses of variance-ANOVA with post-hoc multiple comparisons using the Tukey-Kramer multiple comparisons test for paired parametric comparisons). (**b**) WB for SDF-1α on HL-1 cell lysate following staurosporine treatment.

Since our experimental hypothesis was based on the important role of the CXCR4-SDF-1α axis in cardiac repair, we empirically assessed the SDF-1α levels into myocardial tissue at different time-points following myocardial I/R injury (Figure A2). The chemokine was picked at three h after reperfusion; this time-point was chosen for the in vivo tail vein injection of Exo (Figure 3a). Exo^CXCR4^, but not Exo^CTRL^, improved left ventricular ejection fraction (LVEF) at 7 days (Figure 3b). Both types of Exo significantly improved LVEF at 4 weeks, as compared to PBS-injected control rats (75.0 ± 1.4% and 63.5 ± 2.5% for Exo^CXCR4^ and Exo^CTRL^, respectively, versus 53.7 ± 1.1% in the PBS group; *p* < 0.01 and *p* < 0.05 respectively). Exo^CXCR4^ was significantly more effective than Exo^CTRL^ in this regard (*p* < 0.01). Parallel changes in end-systolic and end-diastolic LV volumes were observed (Figure 3c,d). Hemodynamic measurements showed that Exo^CXCR4^ significantly improved –dp/dt min. Although not statistically significant, a trend toward the dp/dt max improvement was also observed in Exo^CXCR4^ group. Exo^CTRL^ improved –dp/dt min only slightly, as compared to the PBS group (Figure 3e). From LV pressure/volume loops, the time constant of isovolumic LV pressure fall (Tau) properties was significantly reduced in hearts injected with Exo^CXCR4^. The whole set of hemodynamic data indicated a global improved in diastolic relaxation (Figure 3f) for hearts injected with Exo^CXCR4^. Finally, hearts in the Exo^CXCR4^ group displayed smaller scars than those in the Exo^CTRL^ or PBS groups (Figure 4a). There also was a significant reduction of scar size in the Exo^CTRL^ group vs. PBS. Both Exo^CTRL^ and Exo^CXCR4^ increased blood vessel density (Figure 4b) at a similar level. 

### 2.3. Exo^CXCR4^ Is Delivered More Efficiently than Exo^CTRL^ to CM in Ischemic/Reperfused Rat Hearts Ex Vivo

**Figure 5:**
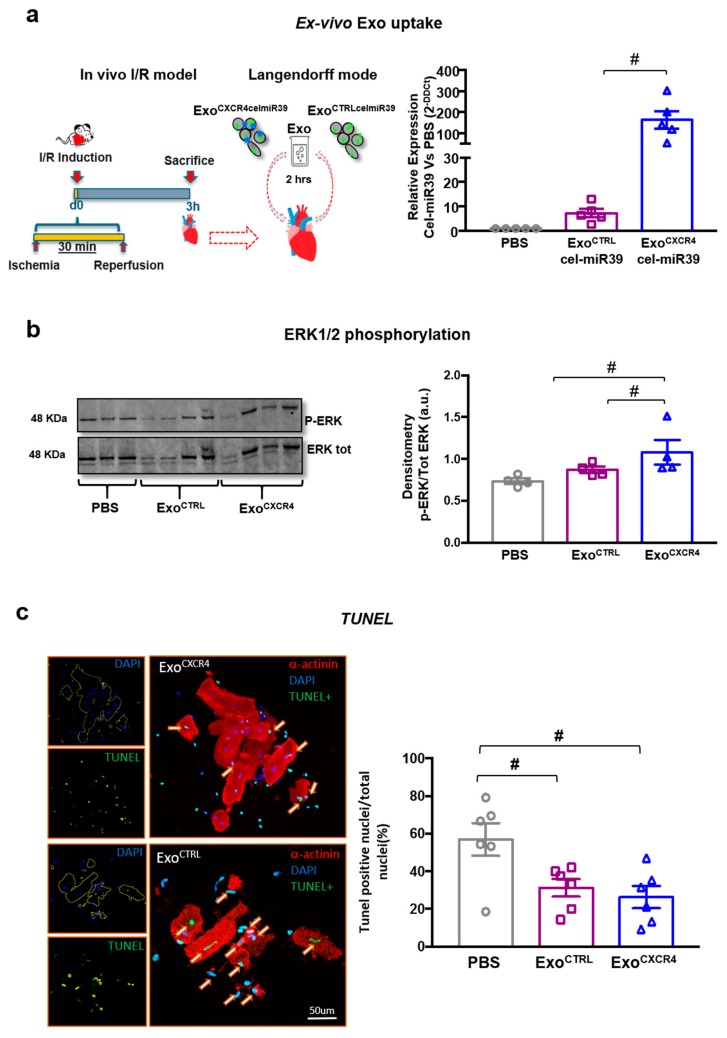
Cellular uptake of Exo by rat adult CM. (**a**) Experimental design. I/R model was induced in rat and 3 h later hearts were excided and retrogradely perfused in a langendorff system for a subsequent 2 h. Exo^CTRLcel-miR39^ and Exo^CXCR4cel-miR39^ were added to the perfusates. PBS was added to the control perfusing solution. (**a**) Exo^CXCR4cel-miR39^ containing perfusates showed increased cel-miR39 levels compared with Exo^CTRLcel-miR39^ and PBS perfused control hearts. Data are 2^−ΔΔ*C*t^ values vs. PBS-perfused hearts (*n* = 5 patients/group) # *p* < 0.05 (two-tailed, paired Student *t*-test). (**b**) Western blotting analyses on cell lysate of isolated adult CM. The levels of ERK1/2 phosphorylation was increased into CM Exo^CXCR4cel-miR39^ from isolated rat hearts. (**c**) TUNEL staining. TUNEL-positive (green nuclei) of apoptotic CM (α-SA -positive cells; red). The α-SA mask is used to detect nuclei within CM on DAPI fluorescence. Quantitative data are expressed as number of green on total CM nuclei, # *p* < 0.05 (one-way analyses of variance-ANOVA with post-hoc multiple comparisons using the Tukey-Kramer multiple comparisons test for paired parametric comparisons).

To assess whether the superior in vivo cardioprotective effect of Exo^CXCR4^ is mediated by increased cardiac homing as a result of SDF-1α upregulation in the injured heart, rat hearts were subjected to in vivo I/R injury. Three hours later, hearts were harvested and perfused retrogradely in a Langendorff-mode (Figure 5a). Equal amounts of Exo^CXCR4celmiR39^ or Exo^CTRLcelmiR39^ (see Section 4) were added to the perfusates in the two groups, and hearts were continuously perfused for 2 h. CM were then isolated from hearts perfused ex vivo, and cel-miR39 levels were determined in the isolated CM as a measure of Exo delivery. Exo^CXCR4celmiR39^ perfused hearts showed increased cel-miR39 levels compared with both Exo^CTRLcelmiR39^ and PBS-perfused control hearts (fold-increase vs. PBS: 162.6 ± 42.6 and 7.7 ± 1.7 for Exo^CXCR4celmiR39^ and Exo^CTRLcelmiR39^ respectively; Figure 5b). In addition, hCXCR4/c-Myc mRNA was detectable in adult CM from hearts perfused with Exo^CXCR4celmiR39^, but not in those receiving Exo^CTRLcelmiR39^ (Figure 5c). These results indicate that following in vivo I/R injury Exo^CXCR4^ is delivered to CM in injured hearts more efficiently than Exo^CTRL^.

### 2.4. Exo^CXCR4^ Shows Superior Ex Vivo Cardioprotective Effects Compared with Exo^CTRL^

Having previously showed that Exo act mostly through MAP kinases pathway [6], we evaluated whether ERK1/2 phosphorylation was improved in adult CM isolated from ex vivo experiments. CM isolated from infarcted and perfused hearts were lysed for WB analysis. Exo^CXCR4celmiR39^ was able to significantly induce ERK1/2 phosphorylation after 2 h of perfusion as compared to PBS perfused hearts (Figure 5b), whereas Exo^CTRLcelmiR39^ was less effective in this regard.

To confirm cytoprotective role of Exo a subset of isolated adult CM were plated on glass coverslip and soon after fixed for TUNEL staining. Both Exo^CTRLcelmiR39^ and Exo^CXCR4celmiR39^ increased the number of surviving CM after 2 h of perfusion as compared to PBS group. The number of adult CM undergoing apoptosis was lower in Exo^CXCR4^ than Exo^CTRL^, although not statistically significant (Figure 3c).

### 2.5. Exo^CXCR4^ Shows Superior In Vitro Cardioprotective Effects Compared with Exo^CTRL^

**Figure 6:**
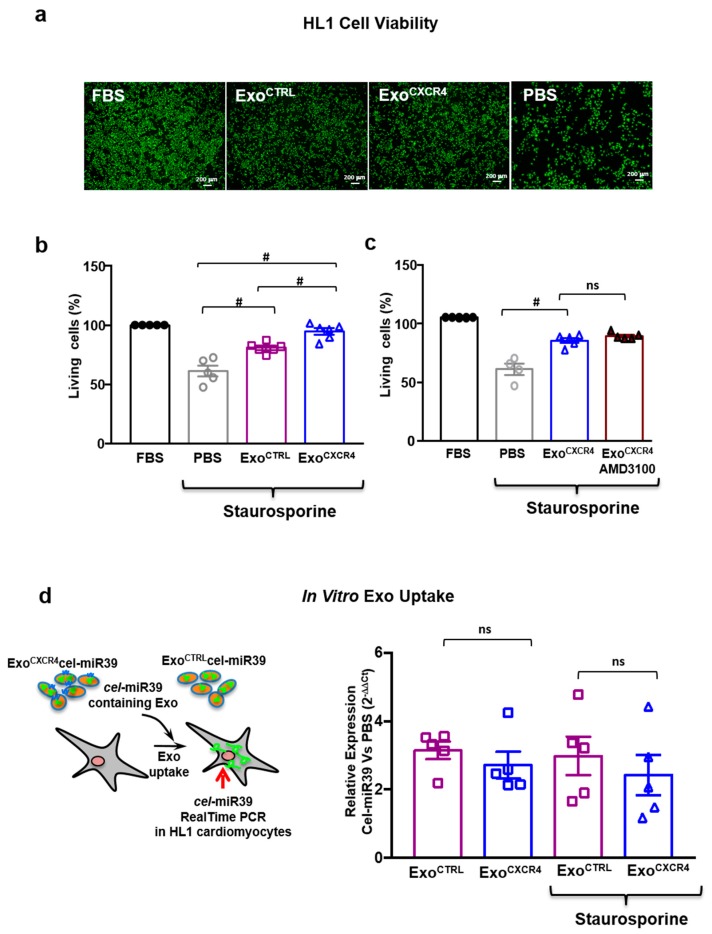
In vitro bioactivities of Exo^CXCR4^. (**a**,**b**) Exo^CTRL^, and Exo^CXCR4^ inhibit staurosporine-induced death in HL-1 CM (green) (*n* = 6 patients/group for Exo^CTRL^ and Exo^CXCR4^; *n* = 5 repeated measurements for PBS and FBS) # *p* < 0.01 (one-way analyses of variance-ANOVA with post-hoc multiple comparisons using the Tukey-Kramer multiple comparison tests for paired parametric comparisons). Exo^CXCR4^ exhibit superior pro-survival effect. (**c**) Exo^CXCR4^ superior pro-survival effect in HL-1 CM is not affected by the addition of AMD3100 as specific CXCR4 antagonist. (**d**) In vitro Exo uptake. Real-time PCR analyses of cel-miR39 (mature sequence) levels in HL1 CM incubated with Exo^CTRLcel-miR39^ and Exo^CXCR4cel-miR39^ during normal or stress conditions. Data are 2^−ΔΔ*C*t^ values vs. PBS (*n* = 5 patients/group).

**Figure 7:**
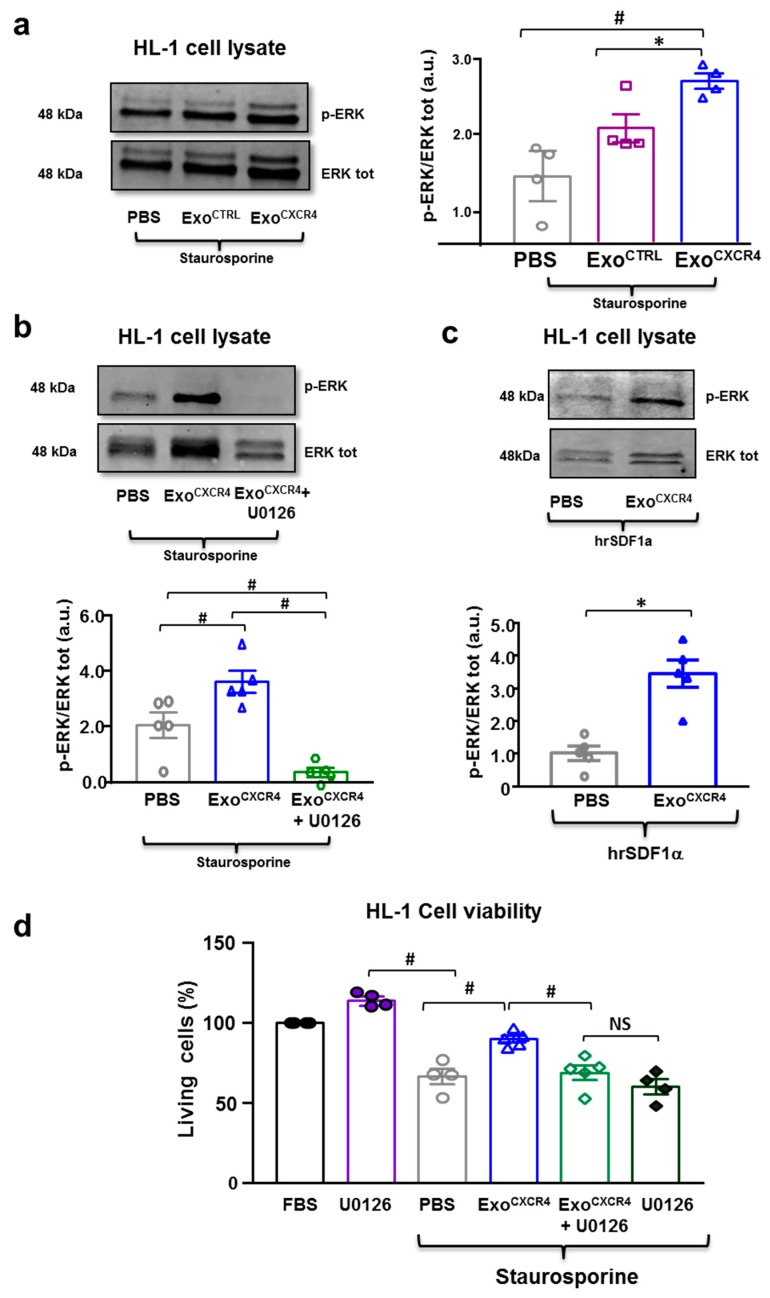
(**a**) Western blot showing pro-survival ERK1/2 phosphorylation in HL-1 CM when Exo^CTRL^, and Exo^CXCR4^ were added to the medium in stress-conditions (*n* = 4 patients/group) # *p* < 0.01 (Kruskal-Wallis test nonparametric multiple comparisons with Dunn’s multiple comparison test); * *p* < 0.05 (two-tailed, paired Student *t*-test). (**b**) ERK1/2 phosphorylation is prevented by adding the kinases chemical inhibitor U0126. (*n* = 5 patients/group for Exo^CTRL^ and Exo^CXCR4^; *n* = 5 repeated measurements for PBS) # *p* < 0.01 (one-way analyses of variance-ANOVA with post-hoc multiple comparisons using the Tukey-Kramer multiple comparisons test for paired parametric comparisons). (**c**) Human recombinant SDF-1α is able to stimulate CXCR4 receptor in mouse HL-1 CM only after these cells have uptaken the human receptor from Exo (*n* = 5 patients/group); * *p* < 0.05 (two-tailed, paired Student t-test). (**d**) Exo ^CXCR4^ are significantly less effective in preventing staurosporine cell death when HL1 CM are treated with the kinases chemical inhibitor U0126, (*n* = 5 patients/group for Exo^CXCR4^ and Exo^CXCR4^+U0126; *n* = 4 repeated measurements for PBS ) # *p* < 0.05 (one-way analyses of variance-ANOVA with post-hoc multiple comparisons using the Tukey-Kramer multiple comparisons test for paired parametric comparisons). Cell vitality was not affected by U0126 alone (*n* = 4 repeated measurements).

**Figure A3:**
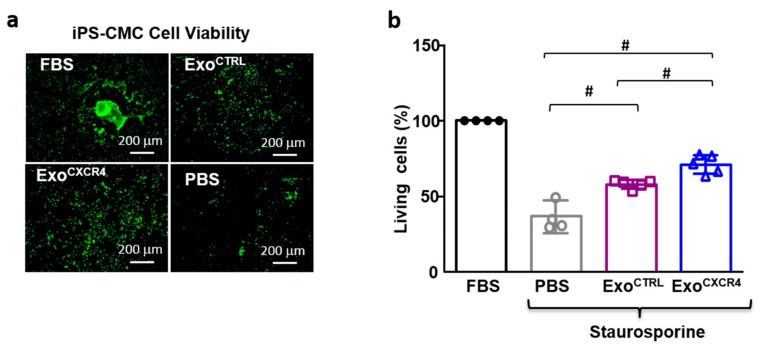
In vitro bioactivities of Exo^CXCR4^. (**a**) Exo^CTRL^, and Exo^CXCR4^ inhibit staurosporine-induced death in human iPS-derived CM (**b**) (*n* = 5 patients/group for Exo^CTRL^ and Exo^CXCR4^; *n* = 4 repeated measurements for PBS and FBS) # *p* < 0.05 (one-way analyses of variance-ANOVA with post-hoc multiple comparisons using the Tukey-Kramer multiple comparisons test for paired parametric comparisons).

**Figure A4:**
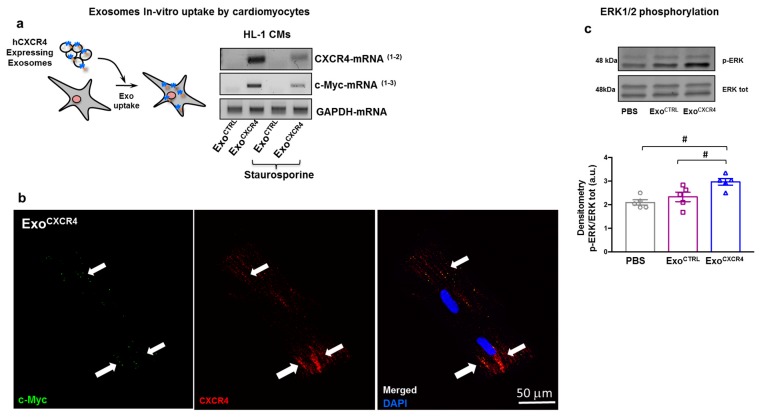
In vitro transfer of CXCR4 mRNA and protein into target cells. (**a**) The c-Myc-mRNA sequence is transferred by Exo into target cells in both physiological and stress conditions (staurosporine). (**b**) Immunofluorescence staining showing the presence of c-Myc-tag protein (white arrows) into adult CM isolated from heart perfused ex vivo with Exo^CXCR4cellmiR39^. For the wild-type CXCR4, protein cells have been stained with a primary anti-CXCR4 antibody, which recognizes human and rat protein. (**c**) Phosphorylation of ERK1/2 is enhanced in HL-1 CM after treatment with Exo^CXCR4^ (*n* = 5 patients/group for Exo^CTRL^ and Exo^CXCR4^; *n* = 5 repeated measurements for PBS ) # *p* < 0.05 (one-way analyses of variance-ANOVA with post-hoc multiple comparisons using the Tukey-Kramer multiple comparisons test for paired parametric comparisons).

Exo^CTRL^ is cytoprotective in neonatal HL-1 [6,7]; here we compared its cytoprotective activity with that of Exo^CXCR4^ using the in vitro model of staurosporine-induced death in HL-1 cardiomyocytes. Exo^CXCR4^ was more effective than Exo^CTRL^ in preventing staurosporine-induced death in HL-1 cells (Exo^CXCR4^ 94.7 ± 2.6%; Exo^CTRL^ 80.9 ± 1.8%) (Figure 6a,b). Similar results were obtained in human iPSC-derived CM, as Exo^CXCR4^ was more effective than Exo^CTRL^ at preventing staurosporine-induced death in these cells (Exo^CXCR4^ 73.0 ± 2.7%; Exo^CTRL^ 58.6 ± 1.2%; Figure A3). Furthermore, the effect of Exo^CXCR4^ in HL-1 cells was not antagonized by ADM3100, indicating that is was independent from SDF-1α (Figure 6c). Similar intracellular levels of celmiR39 was observed in normal or stress conditions when Exo^CXCR4celmiR39^ and Exo^CTRLcelmiR39^ were added to the media of HL-1 cells; indicating that the presence of CXCR4 on Exo surface did not increase in vitro uptake (Figure 6d). This finding is probably related to the absence of an SDF-1α gradient in this in vitro model, as opposed to the in vivo situation, as shown by WB in vitro analysis (Figure A2b). Both Exo^CTRL^ and Exo^CXCR4^ induced ERK1/2 phosphorylation after staurosporine-induced damage. Compared with Exo^CTRL^, Exo^CXCR4^ treated HL-1 showed higher levels of phosphorylated ERK1/2 in both staurosporine-induced (Figure 7a) and normal (Figure A4c) conditions. The effect was prevented by adding the kinases chemical inhibitor U0126 (Figure 7b). In parallel, U0126 was able to abolish Exo^CXCR4^ protective effect on HL-1 CM (Figure 7d). This finding is in line with our previous data [6], showing that the ERK pathway is involved in the anti-apoptotic effect mediated by Exo.

### 2.6. Exo^CXCR4^ Transfers CXCR4 mRNA and Protein into CM In Vitro

We hypothesized that the superior cytoprotective in vitro effect of Exo^CXCR4^ was due to the transfer of bioactive CXCR4 to target cells that may enhance the previously described cytoprotective effect of Exo (which also includes PAPP-A mediated protease activity) [6]. We tested the presence of exogenous hCXCR4 in murine HL-1 CM at mRNA and protein levels. The hCXCR4-mRNA was detected in HL-1 CM exposed to Exo^CXCR4^ but not in those exposed to Exo^CTRL^. Moreover, the c-Myc nucleic acid sequence within the hCXCR4-c-Myc construct was detected by RT-PCR in cells exposed to Exo^CXCR4^ but not in those exposed to Exo^CTRL^ (Figure A4a). This observation was made both under basal conditions and after staurosporine-induced cell stress (Figure A4a). The c-Myc tag was detectable at the protein level in CM treated with Exo^CXCR4^, demonstrating the translation of the hCXCR4-c-Myc fusion construct, but not in those receiving Exo^CTRL^ (Figure A4b). These results demonstrate the ability of Exo^CXCR4^ to deliver the mRNA encoding for the exogenous hCXCR4, which may result in the translation of hCXCR4 protein in mouse CM. Because hCXCR4 bioactivity requires the presence of SDF-1α [18], we used a species-specific human recombinant SDF-1α protein (hDF-1α) that does not activate the downstream ERK signal in mouse HL-1 cells in order to assess hCXCR4 bioactivity in these cells (Figure 7c). HL-1 cells pre-treated with Exo^CXCR4^, but not pre-treated with Exo^CTRL^, exhibited increased ERK1/2 phosphorylation after the subsequent exposure to hDF-1α (Figure 7c). These results indicate that Exo^CXCR4^ delivers bioactive hCXCR4 to target cells.

## 3. Discussion

Exo have emerged as a therapeutic tool with the potential to replace cellular approaches in the treatment of cardiovascular disease [6,7,27,28]. Compared with cell therapy, Exo offer several potential advantages, as they can be stored at −80 °C without a substantial loss of functional activity [6,7,29], they are effective after systemic delivery [30], with a dosage that is not limited by microvascular plugging [31], and they are poorly immunogenic [32]. With respect to therapeutic applications, safety and efficacy of Exo depend on their bio-distribution that influence selective and effective uptake, respectively, by target cells after systemic delivery [33]. In fact, a given Exo is taken up with different efficiencies by different recipient cell types [34]. Systemically delivered Exo is largely up taken by the liver, and to lesser extents by spleen and lungs [35,36,37], although in vivo Exo bio-distribution also depends on the producing cell type [12]. In animal models of ischemic heart disease, IM Exo administration was beneficial in several studies [6,7,8], whereas IC Exo administration was tested in a single study [15]. The latter found that IM delivered Exo from CPCs, but not IC delivered Exo from the same producing cells were beneficial in a pig MI model, mainly due to comparatively lower Exo retention in the heart using the IC route of administration. From a translational perspective, IM Exo injection would hardly be feasible for treating acute MI patients, thus, improving Exo retention by the heart after IC or systemic administration is a crucial goal. Recently studies have attempted to engineer transmembrane proteins of Exo using “cardiac-homing peptide” to increase targeting capability to cardiac tissues [38,39,40]. These study showed that homing peptides displayed on the external part of Exo, although not directly implicated into mechanism of action of Exo, promote the specificity and efficiency of systemic delivery of Exo.

Our experimental approach leverages on the important role of the CXCR4-SDF-1α axis in cardiac repair. SDF-1α is markedly upregulated within 1 h of myocardial ischemia, resulting in a chemoattractant gradient for CXCR4 expressing leukocytes and stem cells [41]. Within the heart, SDF-1α expression was demonstrated for both CM and fibroblasts a few hours after acute MI [41]. In light of the above, we hypothesized that beside the physiological ischemia-induced cardiac SDF-1α expression, overexpressing CXCR4 in Exo would result in an enhanced homing of Exo to the injured myocardium, and thus, increased cardioprotective effects. To test this hypothesis, we genetically modified CPCs to overexpress CXCR4 and then isolated the secreted Exo. CXCR4 was detectable on Exo^CXCR4^ but not on Exo^CTRL^ released from naive CPCs. Exo homing to, and uptake by, CM was studied ex vivo in isolated-perfused hearts after in vivo I/R injury. Exo^CXCR4^ were markedly more effective than Exo^CTRL^ in this regard. It should be emphasized that Exo uptake was specifically studied in CM isolated from ischemic rat hearts, as opposed to the total heart cell population. We did not evaluate and discriminate Exo uptake by other cell types within the ischemic heart, such as fibroblast and endothelial cells. This represents a limitation of the study, however, we have evidence showing that the benefit of Exo^CXCR4^ and its superior effect as compared to Exo^CTRL^ results in an increased number of surviving cardiomyocytes (Figure 4a and Figure 5c) rather than improved angiogenesis (Figure 4b), for which we did not find any significant difference between the groups. Exo uptake measured here includes both homing to I/R heart tissue and CM binding and uptake. These different components of the global “uptake” process cannot be discriminated. For instance, enhanced Exo homing to injured myocardium would increase Exo bioavailability in the extracellular compartment, and therefore on the CM surfaces, and result in an increase in total Exo uptake by CM, even in the case when the intrinsic Exo uptake efficiency (i.e., the intrinsic efficiency of the process at a single particle level) was unaffected. In vivo, we similarly found superior functional benefits in terms of scar size as well as LV systolic and diastolic function for Exo^CXCR4^ compared with Exo^CTRL^. Efficient Exo^CXCR4^ homing to I/R myocardium, as demonstrated in ex vivo isolated-perfused hearts, most likely played an important role in vivo, too. 

Although the increased concentration of SDF-1α cytokine within the heart tissue may enhance bio-availability of Exo in close proximity of CM surface, it does not have a direct effect in mediating the uptake in vitro, where the experimental conditions failed to recapitulate in vivo cytokines gradient (Figure 6a). Indeed, the use of a specific CXCR4 antagonist (AMD3100) has no effect on increasing Exo uptake, neither inhibits protection, in vitro. On the other hand, the transfer of active and SDF-1α-responsive receptor from Exo to target cells, as shown in the Figure 7c, plays a crucial role leading to activation of protective signaling pathways, and in addition is of the already described mechanisms via PAPP-A–mediated IGF-1 release [6]. As expected, the degree of Exo-mediated improvement of cardiac function, previously shown by our group upon IM injection into infarcted hearts [6], could not be matched by the tail-vein injection, likely due to larger in vivo biodistribution. Indeed, at seven days after tail-vein injection of Exo^CTRL^ we did not achieve statistically significant improvement compared to saline group (Figure 7), as was the case by IM injection in previous studies [6,7]. By enhancing homing of Exo to CM via CXCR4 expression on the vesicle surface, we achieved comparable functional improvements using IM and systemic routes of administrations (Figure 3). Previous studies have shown that mesenchymal stem cells (MSCs) overexpressing surface CXCR4 improve migration and engraftment in response to SDF-1α gradients in various disease models [42,43,44]. Taking a methodological approach similar to the one used here, Kang et al. [25] recently showed that Exo secreted from CXCR4 overexpressing MSCs mediated antiapoptotic and proangiogenic effects in vitro, while also indirectly enhancing MSC-mediated CM survival in vivo.

Here, we investigated for the first time the direct effects of systemic Exo^CXCR4^ injection and established Exo homing/uptake as a crucial mechanism by which CXCR4 increases Exo-CPC delivery to CM. Exo^CXCR4^ not only transfer their exogenous protein to the target cells, they are also able to transfer the mRNA, and this confirmed data obtained from different groups [45]. The relative contribution of bioactive CXCR4 versus the transduction of its mRNA by Exo receiving cells has not been detailed in the present study. From the functional point of view, Exo^CXCR4^ have superior in vitro and in vivo anti-apoptotic effects in CM compared to Exo^CTRL^. These results have major translational implications for treatment of acute MI patients, owing to the major advantages associated with systemic drug administration [25], which can be performed upon recanalization of the culprit artery.

## 4. Materials and Methods

### 4.1. Cell Culture

Right cardiac atrial appendage specimens were obtained from patients (*n* = 13) who underwent surgical repair of heart valve disease and gave written informed consent. Protocols used in this study were approved by local Ethical Committee for Clinical Research (Comitato Etico Cantonale, Bellinzona, Switzerland; Rif. CE 2923), and study was performed according to the Declaration of Helsinki. CPCs were derived from atrial specimens, as described [6]. Briefly, atrial specimens were cultured as primary ex vivo tissue explants in basic Iscove’s modified Dulbecco’s medium (IMDM; Lonza, Basel, Switzerland) supplemented with 20% fetal bovine serum (FBS) and 1% *v*/*v* penicillin-streptomycin (Life Technologies, Europe BV, Zug, Switzerland). The cellular outgrowth of atrial explants was collected within 14 days and seeded into fibronectin-coated 25 mL flasks, forming a monolayer of CPCs that were passaged twice by trypsinization before Exo purification from conditioned media. Mouse HL-1 cardiomyocyte cell line (LSU Health Sciences Center, New Orleans, LA, USA) was grown in Claycomb Medium (Sigma, Merck KGaA, Darmstadt, Germany) supplemented with 100 μM norepinephrin, 10% FBS, 1% *v*/*v* penicillin/streptomycin, and 4 mM l-glutamine [46]. AMD3100 (Millipore, Merck KGaA) at the concentration of 10 µM was used as specific CXCR4 antagonist. For experiments performed to establish the bioactivity of human CXCR4 into target cells (Figure 7c), confluent HL-1 were treated with human recombinant SDF-1α chemokine (hDF-1α, Sigma) at the concentration of 100 ng/mL for 40 min. The kinases chemical inhibitor U0126 monoethanolate (Sigma) at the concentration of 20 µM was used to inhibit ERK1/2 phosphorylation; iPS-derived CM were obtained as previously described [45]. Briefly CPC were reprogrammed through Sendai virus infection using OCT3/4, SOX2, KLF4, and MYC (CytoTune™-iPS 2.0 Sendai Reprogramming Kit; Thermo Fisher Scientific, Europe BV, Zug, Switzerland), according to manufacturer’s instructions. Cardiac differentiation of iPSCs was induced using the GibcoTM PSC Cardiomyocyte Differentiation Kit (Thermo Fisher Scientific). Differentiated cells were maintained in RPMI (Gibco, Thermo Fisher Scientific) supplemented with B27 (Gibco) at 37 °C and 5% of CO2 and changed every two days.

### 4.2. CPC Engineering to Overexpress CXCR4

The human sequence of CXCR4 (NM_001008540) was cloned into PCMV3-C-Myc expression plasmid (Sino Biological Inc., Wayne, PA, USA) to encode the hCXCR4 cDNA fused with c-Myc tag under the transcriptional control of a CMV promoter (PCMV3-Cx-Myc). This vector was used to transfect CPCs (CPC^CXCR4^). Control CPC (CPC^CTRL^) were transfected with an empty backbone plasmid. Briefly, CPCs were gently collected by trypsinization and re-suspended in normal growth medium at the density of 1 × 10^5^ cells/mL. The transfection agent Lipofectamine 3000 was diluted in Opti-MEM I medium (both from Thermo Fisher Scientific) and left for 10 min at RT. PCMV3-Cx-Myc plasmid or empty backbone were diluted in Opti-MEM I medium and P3000 reagent (2 µL/µg DNA). After incubation, the mix with Lipofectamine 3000 Reagent was added to the mix, containing the diluted plasmid and incubated for an additional 10 min at RT. The mixture was then gently rocked and incubated at 37 °C, 5% CO2. Two days after transfection, cells were washed several times with PBS and the growth medium was exchanged with Exo-producing medium (DMEM high-glucose, Thermo Fisher Scientific).

### 4.3. Particle Purification

Media conditioned by CPC^CTRL^ and CPC^CXCR4^ were obtained by culturing cells in Exo-producing medium in absence of serum for 7 days [6]. For particle purification, conditioned medium (10 mL) was centrifuged at 3000 *g* for 15 min, filtered through a 0.2 µm membrane (BD Biosciences, Allschwil, Switzerland), centrifuged at 10000 *g* for 15 min, and then ultracentrifuged at 100000 *g* for 4 h. Exo pellets were re-suspended in 100 µL PBS, pH 7.4, and stored at −80 °C. Particle number and size were measured using NanoSight technology (Malvern Instruments, Malvern, UK). 

### 4.4. Flow Cytometry

CPC^CTRL^ and CPC^CXCR4^ were labelled with phycoerythrin (PE)-conjugated antibody anti-CXCR4 (CD184) (eBioscience, Thermo Fisher Scientific) and analyzed by means of direct immunofluorescence with a Cytoflex flow cytometer (Beckman Coulter, Nyon, Switzerland). Isotype-matched, nonreactive fluorochrome-conjugated antibodies were used as controls. Surface antigen markers expressed on Exo were analyzed as described [6]. Briefly, exosomes were incubated with a mix of microbeads coated with anti-CD63, anti-CD9, and anti-CD81 and anti-EPCAM antibody (JSR), and then with fluorochrome-conjugated Abs against CD63, CD9, CD81 (all from BD Biosciences), and anti-CXCR4 (CD184) extracellular (Alomone, Jerusalem, Israel) or anti-CXCR4 intraluminal (Abcam, Cambridge, UK). Beads incubated with in the absence of Exo served as controls. The resulting data were processed using Kaluza™ analysis software (Beckman Coulter, Nyon, Switzerland). 

### 4.5. Immuno-Gold Labeling and Cryo-Electron Microscopy(Cryo-EM)

For cryo-EM, isolated exosomes were resuspended in 30 mM HEPES, pH 7.4, containing 100 mM KCl. Immuno-gold labelling was done by successive incubations of the exosome solution with 1:10 anti-CXCR4 antibody (Millipore) overnight, at 4 °C, then with 1:25 of either 15 and/or 25 nM gold-conjugated goat Anti-Rabbit secondary antibodies (Aurion), for 3 h, at room temperature. A 4–5 µL drop was deposited on glow-discharged 200-mesh copper grids with 2 µm holes (Quantifoil R2/2, Quantifoil Micro Tools, Großlöbichau, Germany), then plunged frozen in liquid ethane using a Leica EM-GP (Leica Microsystems, Wetzlar, Germany), at 90% humidity. For examination, all grids were transferred under liquid nitrogen to a Gatan 626 cryo-holder (Gatan). Imaging was done under low-dose conditions (~10 e- per Å2) by means of a 4k × 4k Ceta camera, on a FEI Talos 200C S/TEM (FEI Company), operated at 200 keV. Images were acquired at nominal magnifications of 36,000× and 45,000×, and at 3 to 5 µm underfocus, giving a final object sampling of 4.1 and 3.2 Å per pixel, respectively.

### 4.6. Protein Isolation and Western Blot Analyses

Total proteins were extracted by lysing Exo or cells with ice-cold RIPA buffer (25 mM Tris pH 7.4, 150 mM NaCl, 1 mM EDTA, 1% Igepal CA 630, 1% Na-deoxycholate, 0.01% sodium dodecyl sulfate; SDS) supplemented with protease inhibitors (SIGMAFAST™ Protease Inhibitor Tablets; Sigma) for 30 min at 4 °C under agitation. Exosome and cell lysates were centrifuged at 9500 g for 15 min at 4 °C, and protein concentrations were determined using the BCA kit (Sigma). Proteins were boiled with Laemmli SDS sample buffer 6× (0.375M Tris-HCl pH 6.8, 12% SDS, 60% glycerol, 0.6 M DTT, 20% (*v*/*v*) β-mercaptoethanol, 0.2% (*w*/*v*) bromophenol blue; VWR International, Dietikon, Switzerland), separated on 4–20% Mini-PROTEAN® TGX™ Precast Gel (Bio-Rad Europe, Basel, Switzerland), and transferred onto a PVDF membrane with a semi-dry transfer system (Bio-Rad Europe). The membranes were first blocked for 1 hr in Odyssey Blocking Buffer (LI-COR Biosciences, Bad Homburg vor der Höhe, Germany) diluted 1:1 in distilled water and supplemented with 0.2% Tween 20 (OBB-T), then incubated with the appropriate primary Ab diluted in OBB-T at 4 °C overnight under gentle agitation. The membranes were rinsed and then incubated with an IRDye^®^ 680RD or 800CW goat anti-mouse or goat anti-rabbit secondary Ab (LI-COR Biosciences; 1:15000 dilution in OBB-T) at RT for 2 h. The infrared signal was detected using the Odyssey CLx Detection System (LI-COR Biosciences). Anti-TSG101 rabbit monoclonal Ab (1:1000) was from Abcam; anti-CXCR4 rabbit polyclonal Ab (1:1000) was from Millipore, anti-β-actin mouse monoclonal HRP-conjugated Ab (1:25000) was from Sigma, anti p-ERK, anti total ERK rabbit polyclonal Ab (1:1000) were from Cell Signalling, anti-SDF1-α rabbit polyclonal (1:1000), and anti-GAPDH rabbit polyclonal (1:1000) were from Abcam. For the evaluation of ERK1/2 phosphorylation in adult CM isolated from ex vivo perfused hearts, total proteins were extracted by lysing adult CM with ice-cold RIPA buffer supplemented with protease inhibitors for 30 min at 4 °C under agitation. Western Blot analysis was performed using the protocol described previously. Antibodies anti p-ERK, anti-total ERK rabbit polyclonal Ab (1:1000) were used. The homodimeric active form of PAPP-A was specifically measured by dot immunoblotting under non-denaturing conditions using mouse monoclonal 4PD4-PAPP30 antibody (HyTest, Turku, Finland).

### 4.7. In Vitro and Ex Vivo Exo Uptake by CM

To assess in vitro and ex vivo cellular uptake of Exo by CM, both CPC^CXCR4^ and CPC^CTRL^ were transfected with a pre-miRNA precursor for cel-miR-39 (Thermo Fisher Scientific), a C. elegans miRNA not present in mammalian cells. Intracellular cel-miR39 levels can be used as a measure of cellular uptake of cel-miR39 containing exosomes. Exo released from these cells are referred hereby as Exo ^CXCR4celmiR39^ (from CPC^CXCR4^) and Exo ^CTRLcelmiR39^ (from CPC^CTRL^). In vitro HL-1 were incubated with Exo ^CXCR4celmiR39^ or Exo ^CTRLcelmiR39^. ^CTRLcelmiR39^ in normal conditions or after treatment with staurosporine. Ex vivo isolated rat hearts were perfused in a Langendorff-mode using perfusates supplemented with Exo ^CXCR4celmiR39^ or Exo ^CTRLcelmiR39^, and cel-miR-39 levels were measured in HL-1 and primary adult rat CM using real-time RT-PCR. The level of expression cel-miR39 in the CPC or Exo was consistent with dose-response efficiencies of transfection [6]. 

### 4.8. Levels of Exogenous mRNA into CM

PCR was performed using 2 pairs primers that specifically amplified the hCXCR4 sequence only or the hCXCR4 sequence fused with c-Myc tag (1,2 for human CXCR4-mRNA; 1–3 c-Myc-mRNA; see scheme in Figure A1). The murine sequence of CXCR4 receptor was not amplified by primers (Figure A4), thus allowing the identification of mRNA carried by human derived Exo (hCXCR4-mRNA), or overexpressed CXCR4 (c-Myc-mRNA).

### 4.9. In Vitro CM Viability Assay

To assess HL-1 CM viability, cells were seeded at a 6.25 × 10^4^ cells/cm^2^ density in 96-well plates. After 24 h, cell death was induced by 1μM staurosporine (Sigma). Exo^CTRL^ or Exo^CXCR4^ (isolated from conditioned media of cells from *n* = 6 patients) were added on top of staurosporine at the 3 × 10^6^ particles/cm^2^ concentration, as assessed by Nanosight [6]. In some of the tested conditions, the role of SDF-1α was tested by adding AMD3100 (10 µM) as CXCR4 specific antagonist. Twelve hours later, cells were stained with Cellstain™ Double Stain Kit (Dojindo EU, München, Germany) for 30 min at 37 °C and counted under a fluorescence microscope.

Functional data were validated in human iPS-derived CM. Cell death was induced in iPS-CM by 1 μM staurosporine (Sigma). Exo^CTRL^ or Exo^CXCR4^ (isolated from conditioned media from *n* = 5 patients) were added on top of staurosporine at the 3 × 10^6^ particles/cm^2^ concentration, as assessed by Nanosight. Twelve hours later, cells were stained with Cellstain™ Double Stain Kit (Dojindo) for 30 min at 37 °C and counted under a fluorescence microscope.

### 4.10. RNA Extraction, Reverse Transcription and Real-Time PCR

Cells or exosomes were lysed with TRI Reagent (Sigma), and chloroform was added following company instructions. The pellet was air-dried, re-suspended in DEPC water, and quantified with NanoDrop™ 2000c (Thermo Fisher Scientific). For real-time amplification of cel-miR-39 sequence, 5ng of specific RNA was reverse-transcribed using the Taqman miRNA reverse stem-loop primers (Applied Biosystems, München, Germany) with a T100 Termal Cycler (Bio-Rad). Real-time PCR for specific cell-miR-39 sequence was performed by adding to 1.33 μL cDNA: 1 μL 20× specific primer (Applied Biosystems), 10 μL TaqMan Universal PCR Master Mix, and 7.67 μL DEPC water. Amplification and detection of specific products were performed in triplicate using a CFX Connect™ Real-Time PCR Detection System (Bio-Rad). Negative controls were included in each RT-PCR assay. The threshold cycle (*C*t) of each gene was automatically defined and normalized to the control miR16 (Δ*C*t value) for CPCs, HL-1 CM and exosomes, and to the control U87 or snoRNU202 for rats primary CM. Data are shown as 2^−ΔΔ*C*t^ values.

For PCR amplification of hCXCR4 mRNA sequences, 500 ng of total RNA was reverse transcribed using the GoScript™ Reverse Transcription System (Promega Madison, Dübendorf, Switzerland). Before use for PCR, reactions samples were diluted 1:2 in DEPC water. PCR was performed by adding to 2 μL cDNA, 4μL specific primer mix (1 µL primer forward:1 µL primer reverse:1 µL DEPC water), 5 μL 5× Green Reaction buffer, 0.5 μL 10 mM dNTPs mix, and 0.125 μL Taq enzime. Samples were incubated at 95 °C for 2 min, 95 °C for 30 s and 60 °C for 30 s, 72 °C for 1 min, for 35 cycles. The following couple of primers were used: endogenous human CXCR4 forward: 5-TCTGTGAGCAGAGGGTCCAG-3; reverse: 5-GCTGGAGTGAAAACTTGAAGACT-3. Exogenous human CXCR4-c-Myc forward 5-TCTGTGAGCAGAGGGTCCAG-3 reverse: 5-TCCTCTTCTGAGATGAGTTTCT-3.

### 4.11. Immunofluorescence and Histology

Cells were fixed with paraformaldehyde 4%, sucrose 2%, permeabilized with 0.5% Triton for 30 min and blocked with 5% serum for 30 min at 25 °C. Samples were then incubated with primary antibodies in 5% serum direct against CXCR4 (Abcam 1/100), c-Myc tag (Abcam 1/200) overnight at 4 °C. After 3× wash with PBS, each 10 min, Alexa Fluor secondary antibodies (Life Technologies, 1/1000) were used for detection. Images were acquired by a confocal microscopy system C2 Plus (Nikon). Harvested hearts were cryo-preserved in optimal cutting temperature (OCT) medium at −80 °C. For morphometric analysis, hearts were cut in 8-µm sections, and stained with Masson- trichrome (HT15 Trichrome Stain Kit; Sigma) for measurement of scar size. On each heart section, scar area was determined by tracing the infarct borders manually using ImageJ software (NIH Image, Bethesda, MD, USA). Six sections distributed at fixed steps from the cardiac basis to the apex were analyzed for each heart and values were averaged. Anti-SMA (1:100) (Santa Cruz, CA, USA), smooth muscle actin, was used to stain vessels and TnI (1:100) (Santa Cruz, Santa Cruz, USA), Troponin I, as marker for CM, on each heart section. The number of vessels and surface area were quantified using ImageJ software. Six sections for each rat were used for the analysis and values were averaged.

### 4.12. SDF-1α ELISA

To assess the amount of SDF-1α protein on heart tissues of infarcted rats compared to control, ELISA Kit for rat SDF1/CXCL12 was used (LS Bio, Seattle, WA, USA). Briefly, samples were directly immobilized on to the wells of the microtiter plate. The detection (primary) antibody was added to the wells for binding to its specific antigen for 1 h at 37 °C. After 3 washes, the Detection (secondary) Ab was added and plate incubated for 30 min at 37 °C. A colorimetric substrate (Extra-sensitive TMB) was used for the assay read-out. The accumulation of the coloured product is proportional to the specific antigen present in each well. The results were quantitated with a microtiter plate reader at 450 nm absorbance.

### 4.13. TUNEL Assay

Adult CM isolated from ex vivo experiments were plated on glass coverslips and fixed with paraformaldehyde 4%, permeabilized with 0.2% Triton for 5 min, and equilibrate at room temperature for 5–10 min with Equilibration Buffer. Samples were then incubated with a solution of Nucleotide Mix and rTdT Enzyme in a humidified chamber for 1 h at 37 °C and immersed in 2× SSC for 15 min at room temperature to terminate the reactions. After three washes to remove unincorporated fluorescein-12-dUTP, cells were incubated with a primary antibody against alpha sarcomeric actinin (1:100) and subsequently with an anti-mouse secondary antibody 568 (1:1000), both from Abcam and with DAPI, and immediately analyzed under a fluorescence microscope. A positive control slide using DNase I was included in each experiment. 

### 4.14. Animal Experiments

Experimental protocols were approved by the Animal Care Committee of Canton Ticino, Switzerland (TI-08-18). All procedures conformed to the Directive 2010/63/EU of the European Parliament. A model of myocardial ischemia with reperfusion was used. Ischemia Reperfusion (I/R) was induced in healthy male Wistar rats (250–300 g body weight) anesthetized with a cocktail of Ketamine (Ketasol 100, 100 mg/kg) and Xylazine (Rompun 2%, 80 mg/kg), intubated and ventilated. The left anterior descending artery was ligated near its origin with a 6-0 prolene suture. The coronary ligature was released after 30 min, and exosomes were injected in the tail-vein 3 h after reperfusion with a total of 300 μL of PBS containing 2 × 10^11^ particles of the following vesicle populations: pool of Exo-CPC^CTRL^ (*n* = 6) and pool of Exo-CPC^CXCR4^ (*n* = 6) or PBS. The chest was then closed, pneumothorax was reduced, and the rats were treated with Meloxicam during postsurgical recovery. Sedated rats underwent transthoracic echocardiography at 7 and 28 days post-MI using the Vevo2100 echocardiography system (VisualSonic System 2100, FUJIFILM VisualSonics, Toronto, Canada) equipped with a 15-MHz linear transducer. Under ECG monitoring of heart rate, 2D images of the hearts were acquired in long-axis views at the level of the greatest LV diameter. LV ejection fraction (LVEF) was measured using Simpson’s analysis. 

Hemodynamic analysis was performed at day 28 using a Millar pressure-volume conductance catheter. Rats were anesthetized with an intraperitoneal injection of ketamine (Ketasol 100, 100 mg/kg) and xylazine (Rompun 2%, 80 mg/kg). The trachea was cannulated, and the animal connected to a positive- pressure volume-controlled rodent ventilator. The catheter was introduced through the right carotid artery into the ascending aorta and then into the LV cavity. After hemodynamics measurements, the animal was sacrificed.

### 4.15. Statistical Analyses

Results are presented as means ± SE of *n* independent experiments or patients from which Exo producing cells were derived. Each single “*n*” is specified in the figure legends. Statistical analyses were performed using InStat, version 3.0 (GraphPad Software, Inc., San Diego, CA, USA). The hypothesis that data came from a normally distributed population was assessed using the Kolmogorov Smirnov normality test. The assumption that data were sampled from populations with identical SDs was tested using the method of Bartlett. The Student *t*-test was used to compare two normally distributed populations. One-way analyses of variance (ANOVA) with subsequent post-hoc multiple comparisons using the Tukey–Kramer multiple comparisons test were used for parametric comparisons. The Kruskal–Wallis test was used for nonparametric multiple comparisons. To perform post-hoc pairwise comparisons following Kruskal–Wallis test, Dunn’s non-parametric multiple comparisons procedure was used. Differences with probability values *p* < 0.05 were considered statistically significant. 

## Abbreviations

hDF-1	Human Recombinant SDF-1
Cryo-EM	Cryo Electron Microscopy
IMDM	Iscove’s Modified Dulbecco’s Medium
Exo-CPC	Exosomes Produced by Cardiac-Resident Progenitor Cells
HIF-1	Hypoxia-Induced Factor 1
Exo	Exosomes
CPC	Cardiac-Resident Progenitor Cells
MSC	Bone Marrow-Derived Mesenchymal Stem Cells
Exo ^CXCR4^	Exosomes Expressing CXCR4
CPC ^CXCR4^	Cardiac-Resident Progenitor Cells that Overexpress CXCR4
FBS	Fetal Bovine Serum
CPC ^CTRL^	Control Cardiac-Resident Progenitor Cells
Exo ^CTRL^	Exosomes Produced by Control Cardiac-Resident Progenitor Cells
SMA	Smooth Muscle Actin
TnI	Troponin I
MI	Myocardial Infarction
CM	Cardiomyocyte
EV	Extracellular Vesicle
IC	Intracoronary
IM	Intramyocardial
I/R	Ischemia/Reperfusion
FC	Flow Citometry
WB	Western Blot
PE	Phycoerythrin
